# Flavonoids isolated from *Tridax procumbens* (TPF) inhibit osteoclasts differentiation and bone resorption

**DOI:** 10.1186/s40659-015-0043-6

**Published:** 2015-09-12

**Authors:** Md. Abdullah Al Mamun, Kamrul Islam, Md. Jahangir Alam, Amina Khatun, M. Masihul Alam, Md. Abdul Alim Al-Bari, Md. Jahangir Alam

**Affiliations:** Department of Genetic Engineering and Biotechnology, Shahjalal University of Science and Technology, Sylhet, 3114 Bangladesh; Department of Anthropology, Shahjalal University of Science and Technology, Sylhet, 3114 Bangladesh; Department of Applied Nutrition and Food Technology, Islamic University, Kustia, 7003 Bangladesh; Department of Pharmacy, Rajshahi University, Rajshahi, 6205 Bangladesh

**Keywords:** Bone resorption, Osteoclast differentiation, Pit formation, TRAP positive cells

## Abstract

**Background:**

The *Tridax procumbens* flavonoids (TPF), are well known for their medicinal properties among local natives. The TPF are traditionally used for dropsy, anaemia, arthritis, gout, asthma, ulcer, piles, and urinary problems. It also used in treating gastric problems, body pain, and rheumatic pains of joints. The TPF have been reported to increase osteogenic functioning in mesenchymal stem cells. However, their effects on osteoclastogenesis remain unclear. The TPF isolated from *T. procumbens* and investigated the effects of the TPF inhibit on osteoclast differentiation and bone resorption activities using primary osteoclastic cells. Osteoclast formation was assessed by counting the number of tartrate resistant acid phosphatase (TRAP) positive multinucleated cells and by measuring both TRAP activities.

**Results:**

The TPF significantly suppressed the RANKL-induced differentiation of osteoclasts and the formation of pits in primary osteoclastic cells. The TPF also decreased the expression of mRNAs related to osteoclast differentiation, including *Trap*, *Cathepsin K*, *Mmp*-*9*, and *Mmp*-*13* in primary osteoclastic cells. The treatment of primary osteoclastic cells with the TPF decreased Cathepsin K, Mmp-9, and Mmp-13 proteins expression in primary osteoclastic cells.

**Conclusion:**

These results indicated that TPF inhibit osteoclastogenesis and pits formation activities. Our results suggest that the TPF could be a potential anti-bone resorptic agent to treat patients with bone loss-associated diseases such as osteoporosis.

## Background

The human bone is a highly dynamic organ that maintains its homeostasis through a delicate balance between the bone-forming osteoblasts and the bone-eroding osteoclasts [1, 2]. The dynamic balance between these two cells types results in bone remodeling. Osteoclasts are multinucleated giant cells that differentiated from cells of hematopoietic monocyte macrophage linage under the presence of two critical factors: the receptor activator of NF-kB ligand (RANKL) and the macrophage monocyte colony-stimulating factor (M-CSF) [2]. Both factors are produced by osteoblasts or stromal cells [2]. Osteoclasts, which are essential in bone homeostasis, play a key role in the development of osteoporosis, inflammatory arthritis, and rheumatoid arthritis (RA), osteoporosis, or low bone mineral density (BMD), is a important risk factor for fracture in older women [3]. Progressive bone destruction in RA involves the abnormal activation of osteoclasts, which is due to interactions with synovial fibroblasts and helper T cells that express the RANKL [4, 5]. Traditional medicine is an important source of potentially useful new compounds for the development of therapeutic agents [6]. Emergence of pathogenic microorganisms that are resistant or multi-resistant to major class of antibiotics has increased in recent years due to indiscriminate use of synthetic antimicrobial drugs [7]. In addition, high cost and adverse side effects are commonly associated with popular synthetic antibiotics such as hypersensitivity, allergic reactions, immunosuppression and are major burning global issues in treating diseases [8]. Hence, recent attention has been paid to biologically active extracts and compounds from plant species used in herbal medicines [9]. It has been proved effective in the treatment of diseases simultaneously mitigating many of the side effects which are often associated with synthetic antibiotics [10]. Positive response of plant based drugs and less or no side effects might lies in the structure of the natural products which reacts with toxins and/or pathogens in such a way that less harm is done to other important molecules or physiology of host. The flavonoids isolated from *Tridaxprocumbens* (TPF) were selected in the present study for evaluation of their anti-bone resorptive activities. The *T. procumbens* is well adapted to the harsh climatic conditions and is well known for their medicinal properties among local natives. Whole plants is made into paste and applied on fresh cuts [11]. In ayurvedic medicine, the TPF is recorded as a hepatic stimulant. The TPF from leaves and root bark are traditionally used for dropsy, anaemia, arthritis, and gout. It is used for the treatment of asthma, ulcer, piles, and urinary problems [12]. The TPF is also used in treating gastric problems, body pain, and rheumatic pains of joints [13, 14], however, no data were found regarding the pharmacological and phytochemical evaluation. The aim of the study was to clarify the TPF inhibits on osteoclasts differentiation and bone resorption activities using primary osteoclastic cells. In this study, osteoclast formation was assessed by counting the number of tartrate resistant acid phosphatase (TRAP) positive multinucleated cells and by measuring both TRAP activities. Present results demonstrated that the TPF inhibited osteoclasts differentiation and TRAP activities.

## Results

### Effect of the TPF on osteoclast formation

**Fig. 1:**
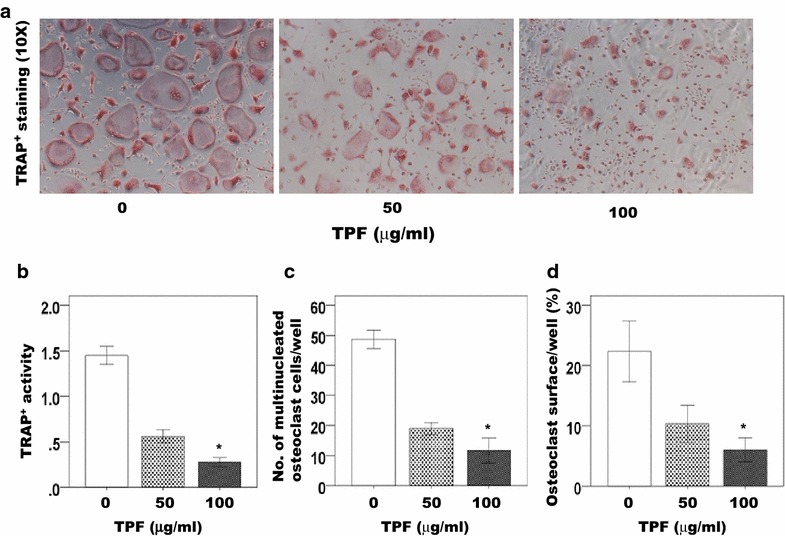
Effect of the TPF on osteoclast formation in vitro. Cells were cultured with RANKL (30 ng/ml) and M-CSF (30 ng/ml) for 7 days in the presence of 0, 50 or 100 μg/ml of the TPF. Osteoclasts were identified via tartrate-resistant acid phosphatase (TRAP). **a** TRAP^+^ cells are shown in *red* and the magnification in **a** represent ×10, **b** TRAP^+^ multinucleated cells characterized by more than three nuclei were counted, **c** osteoclast number/well (N.Oc/well), **d** osteoclast surface/well (Oc.S/well). The data were expressed as the mean ± SD (n = 4) for each group. *p < 0.05

To examine the effect of the TPF on osteoclast formation, cultures of the primary osteoclast cells were treated with the TPF at concentrations of 0, 50 and 100 μg/ml for 7 days. Greater numbers of TRAP-positive multinucleated osteoclasts were observed in the control group in comparison with the TPF treated groups (Fig. 1a, c). Osteoclasts treated with 50 and 100 μg/ml of the TPF were smaller and exhibited fewer nuclei than osteoclasts of the control group (Fig. 1a, c). Moreover, treatment with 100 μg/ml of the TPF resulted in markedly fewer multinucleated osteoclasts in cultures to compare in the control group. Treatment with 100 μg/ml of the TPF strongly suppressed cell–cell fusion among the primary osteoclast cells (Fig. 1a). The TPF inhibited mature osteoclasts in terms of formation of TRAP-positive multinuclear osteoclasts (Fig. 1a–d). TRAP-positive multinucleated osteoclasts activity and osteoclast surface area were decreased significantly in cultures treated with 50 and 100 μg/ml of the TPF than the control group. Treatment with the TPF reduced the number of mature osteoclasts were dose dependent manner (Fig. 1a).

### The TPF inhibited pit formation

**Fig. 2:**
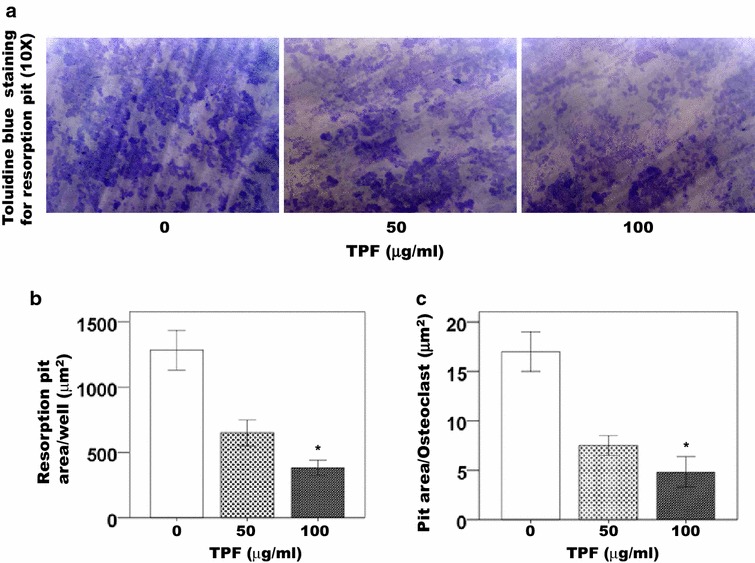
Effect of the TPF on osteoclast activity as indicated by resorption pit area. Cells were cultured with RANKL (30 ng/ml) and M-CSF (30 ng/ml) for 7 days in the presence of 0, 50 or 100 μg/ml of the TPF and discs were fixed and stained with toluidine blue. **a** Effects of the TPF on the bone resorbing activity mature osteoclasts and the magnification in **a** represent ×10, **b** total resorbed pit area/well and **c** resorbed pit area/osteoclasts. The data were expressed as the mean ± SD (n = 4) for each group. *p < 0.05

The TPF inhibited RANKL-induced pit formation and dose dependently reduced pit formation in the primary osteoclast cells at concentrations of 50 and 100 μg/ml of the TPF compare to the control group (Fig. 2a–c). The TPF inhibited mature osteoclasts formation and resorption pit were decreased significantly in cultures treated with 50 and 100 μg/ml of the TPF than the control group. Treatment with the TPF reduced the total resorpted area of mature osteoclasts (Fig. 1a–c).

### Effect of the TPF on mRNA expression level in the primary osteoclast cells

**Fig. 3:**
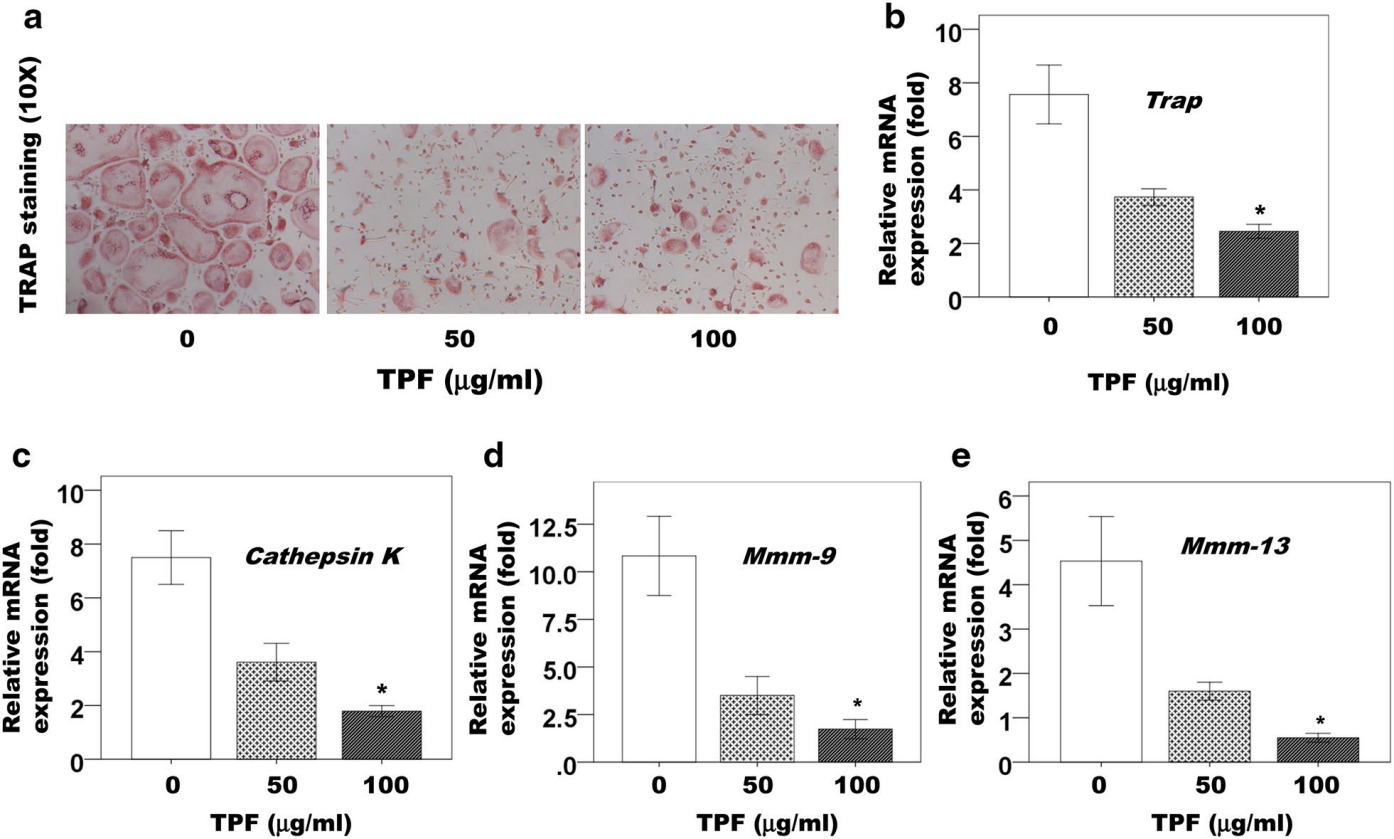
Effect of the TPF on mRNA expression in primary osteoclast cells. **a** TRAP^+^ cells are shown in *red* and the magnification in **a** represent ×10, **b** relative mRNA of *Trap* expression level, **c** relative mRNA of *Cathepsin K* expression level, **d** relative mRNA of *Mmp*-*9* expression level and relative mRNA of *Mmp*-*13* expression level. The data were expressed as the mean ± SD (n = 4) for each group. *p < 0.05

Following treatment with the TPF for 7 days, all cells in each well were harvested; subsequently, cells were used to reverse transcribe mRNA directly into cDNA with the Whole Transcriptase Amplification Kit. The *Trap*, *Cathepsin K*, *Mmp*-*9* and *Mmp*-*13* mRNA levels per well were significantly lower in the TPF-treated cells than in control cells (Fig. 3b–e). Moreover, the *Trap*, *Cathepsin K*, *Mmp*-*9* and *Mmp*-*13* mRNA levels were substantially lower in cells treated with 100 μg/ml of the TPF in comparison with cells treated with 50 μg/ml of the TPF affected The *Trap*, *Cathepsin K*, *Mmp*-*9* and *Mmp*-*13* mRNA levels in a dose dependent manner (Fig. 3b–e).

### Effect of the TPF on Cathepsin K, MMP-9 and MMP-13 activity

**Fig. 4:**
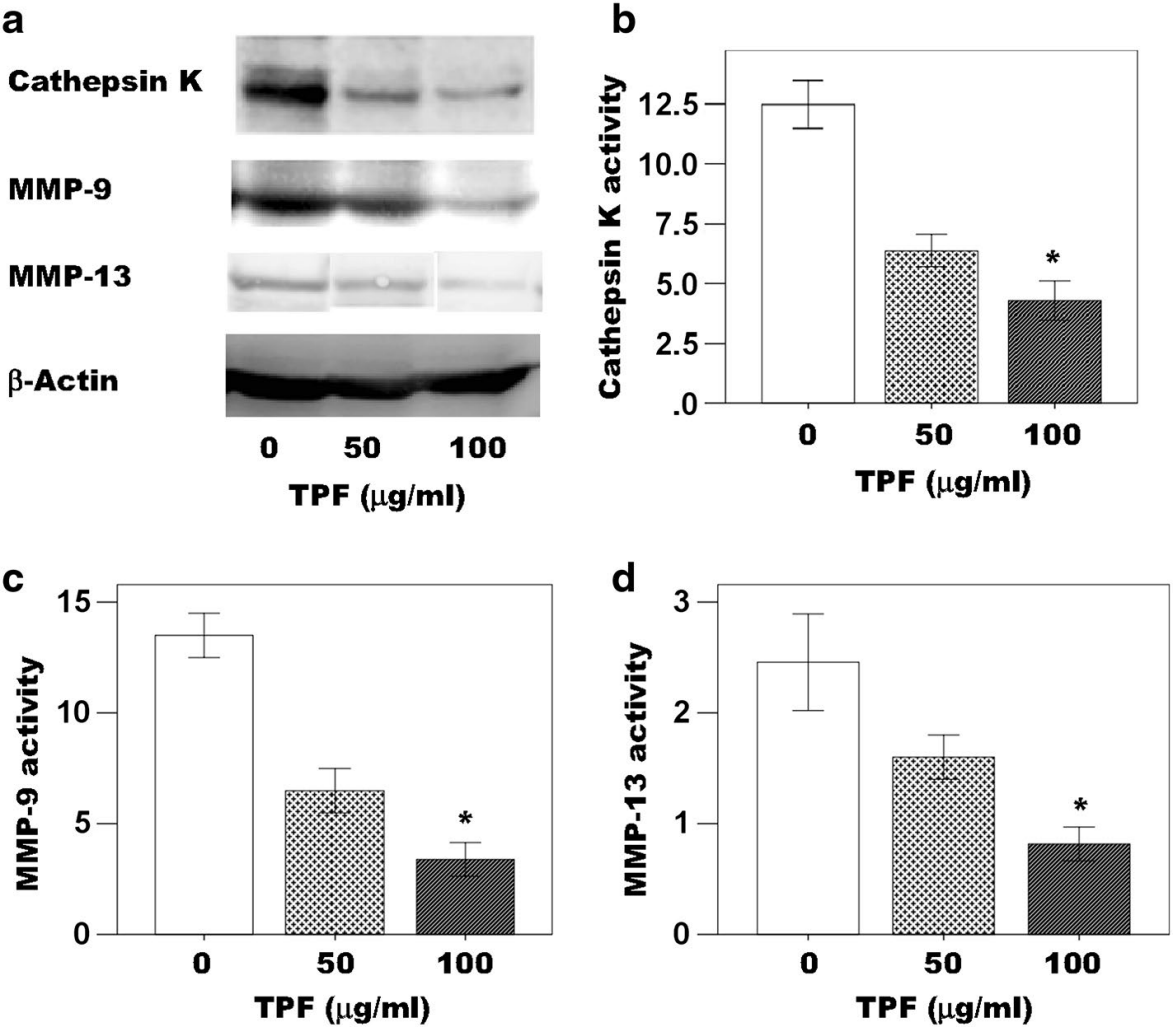
Effects of the TPF on Cathepsin K, MMP-9, MMP-13 and β-actin expression in primary osteoclast cells. **a** The TPF down regulated Cathepsin K, MMP-9 and MMP-13 proteins expression and β-actin served as the control for the protein assay, **b** cathepsin K activity, **c** MMP-9 activity and **d** MMP-13 activity. The data were expressed as the mean ± SD (n = 4) for each group. *p < 0.05

The primary osteoclast cells culture supernatant samples obtained after 7 days in cultures. The pro-Cathepsin K, pro-MMP-9 and pro-MMP-13 activity was detected in all samples (Fig. 4a–d). The Cathepsin K, MMP-9 and MMP-13 samples were diluted with ddH_2_O prior to measurement of enzymatic activity. Pro-Cathepsin K, pro-MMP-9 and pro-MMP-13 activity were significantly lower in those samples derived from cultures treated with 50 and 100 μg/ml of the TPF than in the samples derived from control cultures (Fig. 4a–d). Moreover, The TPF reduced pro-Cathepsin K, pro-MMP-9 and pro-MMP-13 activity in a dose dependent manner (Fig. 4a–d). The concentration of 100 μg/ml of the TPF appeared to suppress pro-Cathepsin K, pro-MMP-9 and pro-MMP-13 activity to a greater extent than the concentration of 50 μg/ml of the TPF.

## Discussion

The present study found that the TPF at doses between 50 and 100 μg/ml significantly inhibited RANKL-induced differentiation of osteoclasts in the primary osteoclastic cells. In doses up to 100 μg/ml of the TPF was not toxic to cultured osteoclast cells (data not shown). It had an inhibitory effect on osteoclasts differentiation of primary osteoclast cells (Figs. 1a–d, 2a–c). Traditional medicines play an important role in health services around the globe. The rational design of novel drugs from traditional medicine offers new prospects in modern healthcare [6, 7, 15]. Dietary habits are known to play a role in the prevention of osteoporosis. Some studies investigated that the potential benefits of fruits, vegetables, tea, and herbs on bone metabolism [15, 16]. It was showed that the consumption of vegetables and fruits inhibited bone resorption in rats better than meat or milk [17]. The meat and milk products commonly believed to be beneficial for bone health [17]. In South Asia, including Bangladesh, India, Pakistan, Nepal and Srilanka, the TPF is made into paste and applied on fresh cuts [11]. Decoctions of the TPF from *T.procumbens* leaves and root bark have been traditionally used for treatment of dropsy, anaemia, arthritis, and gout, ulcer, piles, and urinary problems [12]. The TPF also used in the treatment for fever, typhoid, cough, and diarrhea, gastric problems, body pain, and rheumatic pains of joints [18–20]. TPF has antimicrobial effects such as antibacterial, antifungal and antiviral [21–23]. Recently, our study showed that the TPF can promote the osteoblasts differentiation and bone formation (data submitted). This study investigated the direct effects of the TPF on RANKL-induced osteoclastogenesis in the primary osteoclast cells. The primary osteoclast cells do not contain any osteoblast or bone marrow stromal cells or cytokines [24–26]. The TPF blocked the function of osteoclasts and inhibited osteoclastogenesis and TRAP activity. This inhibition occurred at the stage of osteoclastogenesis when cell fusion and multinucleated cell formation occurs. However, we did not find cytotoxic or cell cycle arrest in the primary osteoclast cells when treated with the TPF at concentrations between 50, and 100 μg/ml (data not shown). Based on the results, the TPF probably blocked osteoclastogenesis by preventing RANKL-mediated activity.

## Conclusion

In summary, this study found that the TPF dose dependently suppressed the osteoclast differentiation in cultured primary osteoclast cells. The regulation of osteoclast differentiation might be an important strategy for the treatment of bone resorption and osteoporosis. The current study demonstrated that the TPF suppress differentiation in primary osteoclast cells. Our results revealed that the TPF may serve as suitable agent in the treatment of bone resorption and osteoporosis.

## Methods

### Samples preparation

*Tridaxprocumbens* plant was identified and authenticated by Professor Dr. Anwarul Islam, Department pharmacy, Rajshahi University, Bangladesh. Samples were collected from the northeast part of Bangladesh and stored at the Plant Biotechnology laboratory, Department of Genetic Engineering and Biotechnology, Shahjalal University of Science and Technology, Sylhet-3114, Bangladesh (Serial no. GEB09032014/3). Different parts of *T. procumbens* (root, stem, leaf, and flowers) were separately shade dried, finely powdered using a blender, and subjected to extraction following the method as described elsewhere, with some modifications [27, 28].

### Reagents

Penicillin, streptomycin, α-MEM, and fetal bovine serum were purchased from Invitrogen (Carlsbad, CA, USA). Recombinant soluble human M-CSF, human RANKL, Recombinant soluble human M-CSF and human RANKL and all other reagents were purchased from Sigma-Aldrich (St. Louis, MO, USA).

### Animals

C57BL/6 male mice were obtained from ICDDRB (Dhaka, Bangladesh) and maintained in our animal care facilities as described elsewhere [29, 30]. The experimental procedures were reviewed and approved by the Animal Care and Use Committee of Shahjalal University of Science and Technology, Sylhet, Bangladesh.

### Osteoclast formation

For the primary osteoclast cells, 8 weeks old female C57BL/6 mice were killed by CO_2_ asphyxia, then the femur and tibia bones were dissected aseptically. The marrow cells were flushed out with α-minimum essential medium (α-MEM, Carlsbad, CA, USA). Bone marrow cells (1.5 × 10^4^ cells/well) were cultured in α-MEM containing 50 ng/ml M-CSF and 50 ng/ml RANKL. The cells were assigned to three groups as follows 0, 50, and 100 μg/ml the TPF. Media were changed on the fourth day. The TPF was added to the appropriate cultures each of the 5 days. After incubation, the cells underwent TRAP staining using a Tartrate Resistant Acid Phosphatase Kit. We observed the TRAP-positive multinucleated cells (with three or more nuclei) under light microscopy and counted them using image analyzing system (KS400; Carl Zeiss, Jena, Germany).

### TRAP activity measurement

After five days culture of bone marrow-derived osteoclasts with appropriate treatment were lysed and incubated for 1 h with a reaction buffer containing para-nitrophenylphosphate (pNPP). The reaction was stopped with 0.3 N NaOH solutions, and optical densities were read and analyzed by microplate spectrophotometer at 405 nm (T60 U, PG Instruments Ltd., England).

### Osteoblasts and osteoclasts co-culture

Eight weeks old female C57BL/6 mice were killed by CO_2_ asphyxia, then the femur and tibia bones were dissected aseptically.  The marrow cells were flushed out with α-minimum essential medium. The bones (without marrow) were cut into pieces (less than 1 mm in diameter), digested with 0.2 % collagenase for 1 h to harvest the mature osteoblast cells. For the osteoclast co-culture cells model, the cells were seeded into 6-wells plates at a density of osteoblasts 2 × 105 cells and bone marrow cells 1.4 × 107 cells/well in α-MEM supplemented with 10 % fetal bovine serum, antibiotics (100 U/ml of penicillin G and streptomycin 100 ng/ml) in a humidified atmosphere with 5 % CO_2_ and 95 % air at 37 °C. The cells were assigned to three groups as follows 0, 50, and 100 mg/ml the TPF. Media were changed on the fourth day. The TPF was added to the appropriate cultures each of the 5 days.  After incubation, the cells underwent TRAP staining using a Tartrate Resistant Acid Phosphatase Kit. We observed the TRAP-positive multinucleated cells (with three or more nuclei) under light microscopy and counted them using image analyzing system (KS400; Carl Zeiss, Jena, Germany).

### Functional bone resorption assay

The primary osteoclast cells were cultured with RANKL (50 ng/ml) and M-CSF (50 ng/ml) for 21 days. Various concentrations of the TPF were added to cultured media to measure osteoclastic cell-mediated mineral resorption. After the culture period, cells were removed using 6 % NaOCl and 5.2 % NaCl. The resorption area was observed under a light microscope and analyzed by using image analyzing system (KS400; Carl Zeiss, Jena, Germany).

### Reverse transcriptase-polymerase chain reaction analysis

Osteoclasts cells were seeded in 6-well plates for 7 days at 37 °C in 5 % CO_2_ in an osteoclast differentiation medium containing 50 ng/ml M-CSF and 50 ng/ml RANKL at a density of 1 × 10^6^ cells/well and cells were treated with the TPF at concentrations of 0, 50, and 100 μg/ml. Total RNA from the cells of each well was isolated using NucleoSpin (Macherey–Nagel, Duren, Germany). RNA aliquots were reverse transcribed to complementary DNAs by using an oligo (dT) primer (Roche), deoxynucleotide triphosphate (dNTP), and Moloney murine leukemia virus (M-MuLV) reverse transcriptase (Fermentas, Hanover, MD, USA). The complementary DNA products were subjected to PCR amplification with gene-specific primers for mouse *Cathepsin K*, *Mmp*-*9*, and Mmp-13 (Table 1). Real-time RT-PCR amplification was performed using a Light Cycler System (Roche) with a Platinum SYBR Green qPCR Super Mix UDG kit (Invitrogen, Carlsbad, CA, USA).

**Table 1:** Primer sequences of real-time PCR

Gene	Forward	Reverse
TRAP	5′CGTCTCTGCACAGATTGCAT	3′AAGCGCAAACGGTAGTAAGG
Cathepsin K	5′CGAAAAGAGCCTAGCGA	3′TGGGTAGCAGCAGAAACA
MM9	5′GAACCAATCTCACCGACAGG	3′GCCACCCGAGTGTAACCATA
MM13	5′GTCTGAGATTTGTAGGCCG	3′TCATCAAGCTTCTGTCTGTGC

### Statistical analysis

We used analysis of variance with an F-test, followed by a t-test. P values less than 0.05 were considered significant. The data are presented as mean ± standard deviation values of independent replicates.
